# Spatial and episodic memory tasks promote temporal lobe interictal spikes

**DOI:** 10.1002/ana.25519

**Published:** 2019-06-20

**Authors:** Umesh Vivekananda, Daniel Bush, James A. Bisby, Beate Diehl, Ashwani Jha, Parashkev Nachev, Roman Rodionov, Neil Burgess, Matthew C. Walker

**Affiliations:** ^1^ Department of Clinical and Experimental Epilepsy UCL Queen Square Institute of Neurology London United Kingdom; ^2^ UCL Institute of Cognitive Neuroscience London United Kingdom

## Abstract

Reflex epilepsies have been demonstrated to exploit specific networks that subserve normal physiological function. It is unclear whether more common forms of epilepsy share this particular feature. By measuring interictal spikes in patients with a range of epilepsies, we show that 2 tasks known to specifically engage the hippocampus and temporal neocortex promoted increased interictal spiking within these regions, whereas a nonhippocampal dependent task did not. This indicates that interictal spike frequency may reflect the processing demands being placed on specific functional–anatomical networks in epilepsy. ANN NEUROL 2019;86:304–309

Reflex epilepsies are characterized by seizures that are provoked by well‐defined cognitive or sensory stimuli, such as primary reading epilepsy and musicogenic epilepsy. It is thought that reflex seizures activate pre‐existing functional–anatomical networks that subserve specific physiological roles (eg, the right temporal region in musicogenic epilepsy^1^). Interictal spikes (IISs) are an electrographic feature of epilepsy that are distinct from seizures, but may define the underlying networks that generate those seizures.^2^ Hence, we asked whether placing processing demands on specific functional–anatomical networks would increase IIS frequency within that network. To address this, we examined intracranial temporal lobe depth electrode recordings from epilepsy patients performing either a spatial memory,^3^ episodic memory,^4^ or attentional bias task.

## Patients and Methods

Two groups of patients with refractory epilepsy undergoing intracranial electroencephalographic (EEG) monitoring for clinical purposes were asked to perform either a spatial (n = 12; 8 with mesial temporal and 4 with extratemporal epilepsy) or episodic memory task (n = 12; 6 with mesial temporal and 6 with extratemporal epilepsy), with a subset of the latter group also performing an attentional bias task on a different day (n = 6; see Table 1). An orthogonal surgical approach was adopted for all patients, who were on antiepileptic medication (either full dose or 50% reduction) at the time of testing. Prior approval was granted by the National Health Service Research Ethics Committee, and informed consent was obtained from each subject. Postimplantation review verified that, for the spatial memory/episodic memory/attentional bias task, 9/11/6 patients had electrode contacts in the hippocampus, 12/12/6 in the lateral temporal lobe, and 9/9/6 in the amygdala.

**Table 1:** Demographics and Epilepsy History of Patients for Spatial Memory Task and Episodic Memory Task

Patient	Age, yr	Gender	Years with Epilepsy	Handedness	Side of Seizure Focus	Region
Spatial memory task						
1	21	F	11	R	R	Temporal
2	28	M	6	R	L	Temporal
3	44	F	21	R	R	Temporal
4	41	M	16	L	R	Hippocampus
5	26	M	13	R	R	Temporal
6	25	M	24	R	L	Occipital
7	22	M	11	R	L	Parietal
8	37	F	16	R	R	Temporo‐occipital
9	30	F	6	R	B	Temporal
10	20	F	14	L	R	Temporal
11	28	M	20	R	R	Temporal
12	29	M	14	R	L	Insula
Episodic memory task						
1	44	F	40	R	R	Temporal
2^a^	22	M	14	R	L	Temporo‐occipital
3	51	F	33	R	L	Temporal
4	23	F	23	A	R	Parietal
5^a^	43	M	39	R	L	Unknown
6	41	F	24	R	R	Frontal
7	42	M	34	R	R	Temporal
8	37	F	10	R	R	Frontal
9^a^	38	M	36	A	L	Hippocampus
10^a^	26	M	20	R	R	Frontal
11^a^	47	F	12	R	R	Temporal
12^a^	38	F	31	R	R	Frontal

a Patients who also performed the attentional bias task.

A = ambidextrous; B = bilateral; F = female; L = left; M = male; R = right.

**Figure 1:**
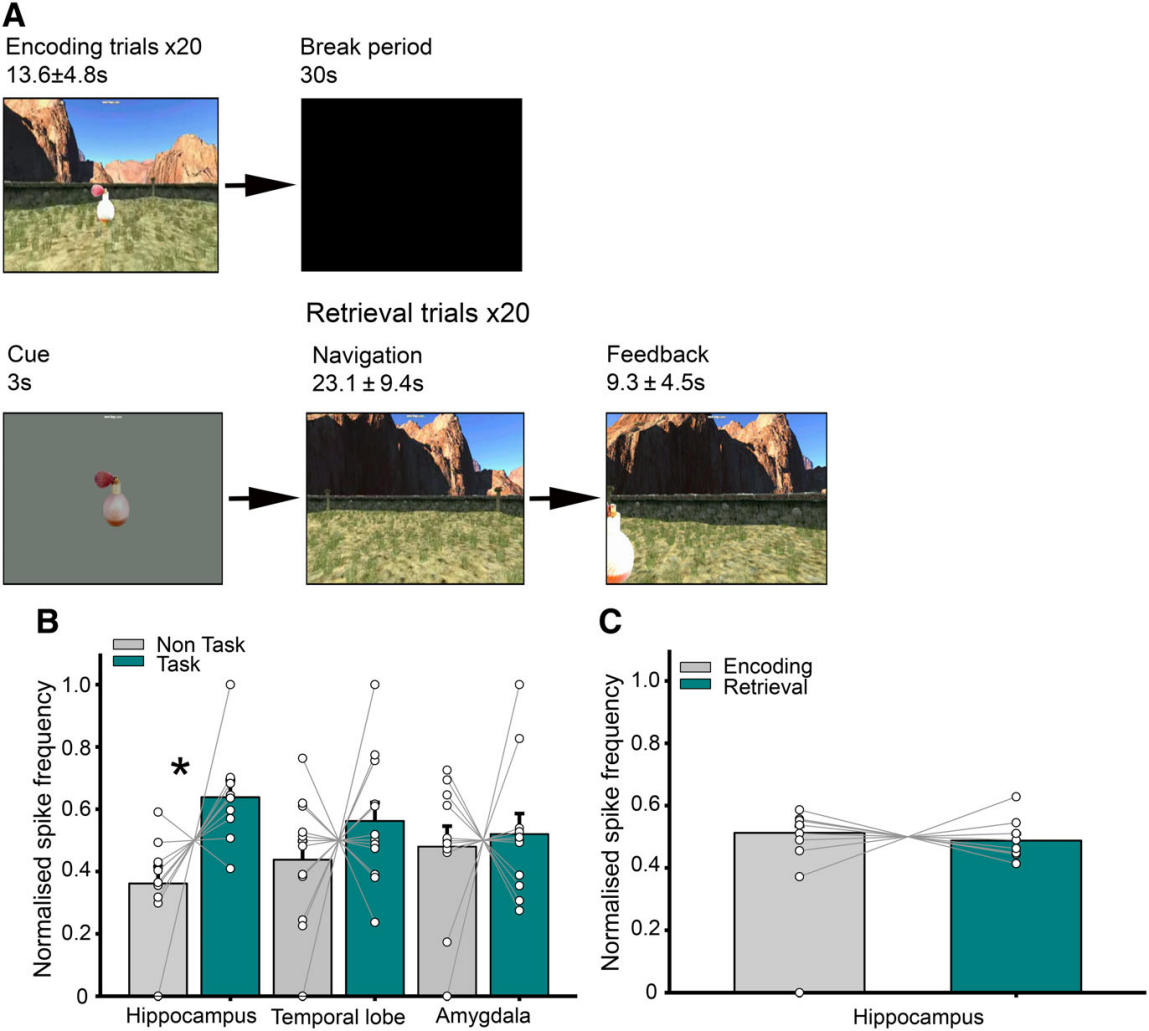
Interictal spike (IIS) frequency is increased in the hippocampus during a spatial memory task. (A) Schematic of the spatial memory paradigm. (B) Normalized IIS frequency for task versus nontask periods. Each line represents 1 patient. (C) Normalized IIS frequency for encoding versus retrieval periods. Asterisk denotes statistical significance. [Color figure can be viewed at www.annalsofneurology.org]

Spatial memory was assessed using a desktop virtual reality environment (Fig 1A; see Bush et al^3^). Patients first navigated toward and memorized the location of 4 objects that sequentially appeared in the environment ("encoding"). Patients were then cued with an image of 1 object ("cue"), placed back in the environment, and asked to navigate toward the remembered location of that object and make a button‐press response ("response"). The object then appeared in its correct location, and the trial ended when they moved to the visible object ("feedback"). The distance error (patient response location vs true location of object) was used as a metric of mnemonic performance.

**Figure 2:**
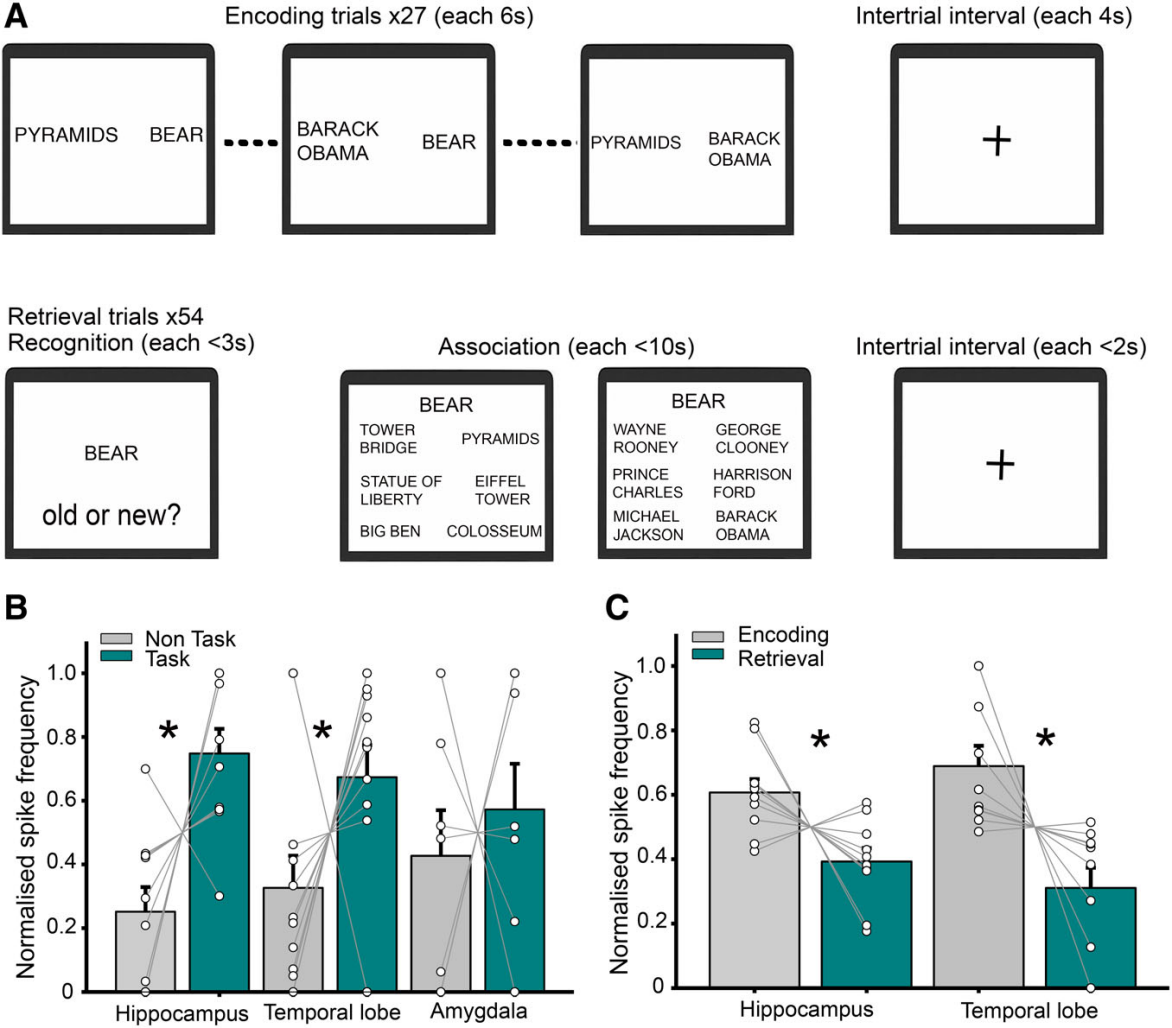
Interictal spike (IIS) frequency is increased in the hippocampus during an episodic memory task. (A) Schematic of the episodic memory paradigm. (B) Normalized IIS frequency for task versus nontask periods. Each line represents 1 patient. (C) Normalized IIS frequency for encoding versus retrieval periods. Asterisks denote statistical significance. [Color figure can be viewed at www.annalsofneurology.org]

In the episodic memory task, patients encoded a number of events, each comprising a location, famous person, and object (Fig 2A; see Horner et al^4^). Each event was encoded across 3 separate trials, where 1 of the pairwise associations comprising that event (ie, object‐location, person‐object, person‐location) was presented, interleaved with pairs from other events. In each trial, patients were asked to imagine the pair of items on screen interacting. Following a short delay, memory for the items and associations from each event was tested, first by asking whether each item was old or new (recognition memory), and second by asking patients to make a 6‐alternative forced choice among potential associates from the same category (associative memory). All associations were tested in both directions on separate retrieval trials, and performance was defined as whether the item or association was correctly identified.

**Figure 3:**
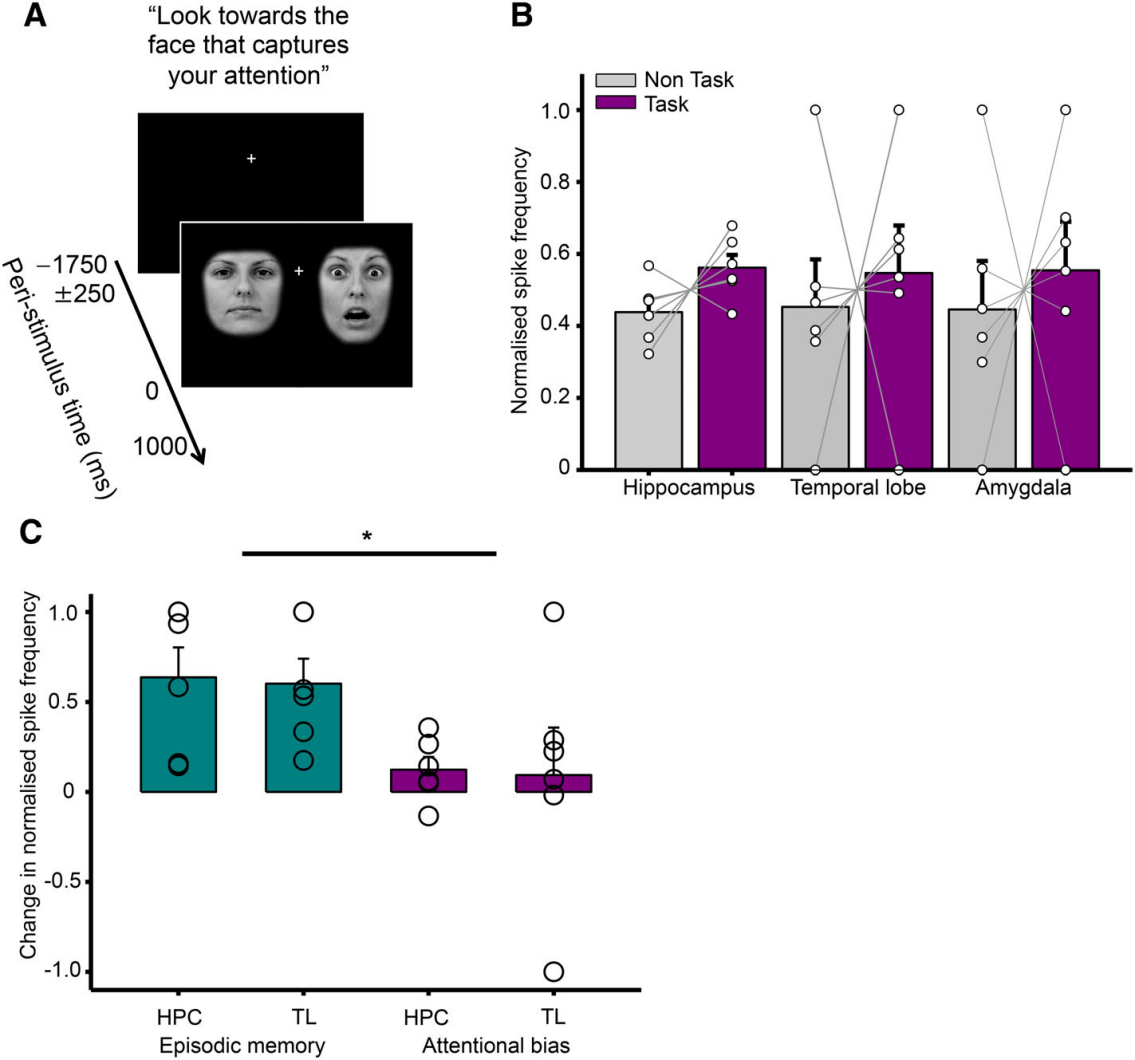
Interictal spike (IIS) frequency is not increased in any region during an attentional bias task. (A) Schematic of the attentional bias paradigm. (B) Normalized IIS frequency for task versus nontask periods. Each line represents 1 patient. (C) Within‐subject task‐related IIS frequency increase compared between episodic memory task and attention bias task. Each circle represents an individual patient. HPC = hippocampus; TL = temporal lobe. Asterisk denotes statistical significance. [Color figure can be viewed at www.annalsofneurology.org]

In the attentional bias task, patients were repeatedly presented with 2 faces placed on either side of a central fixation cross. Each pair of faces comprised 1 neutral and 1 fearful expression performed by the same actor, and patients were simply asked to look toward whichever face captured their attention.^5^ Patients completed 2 to 4 blocks, each of which consisted of 50 trials and lasted ~2 minutes (Fig 3A).

For all patients, IISs were manually identified in the hippocampus, lateral temporal lobe, and amygdala throughout the entire recording session, including both task (which incorporated both encoding and retrieval periods for the memory tasks, excluding intertrial intervals) and interleaved nontask periods of approximately equal duration. Spikes were defined as sharply contoured events that met strict criteria: duration of ≤200 milliseconds, amplitude of >100μV, a subsequent slow wave that disrupted ongoing intracranial EEG activity, and a field distribution consistent with an intracerebral event. In the spatial memory task, retrieval was composed of cue, response, and feedback periods; in the episodic memory task, it was composed of recognition and associative memory test periods. A mean IIS frequency was calculated for each period and region, and IIS frequency differences between periods A and B were normalized to account for between‐subject variance using the formula (A − B) / (A + B).

Finally, to examine the relationship between IIS frequency and performance in the 2 memory tasks, we performed linear regression across trials separately for each electrode contact. Beta coefficients were then averaged across all electrode contacts in each region for each patient, allowing 1‐sample *t* tests at the second level to be performed.

## Results

### *Hippocampal Interictal Spike Frequency Is Increased during a Spatial Memory Task*

First, we compared normalized IIS frequency during the spatial memory task with nontask periods immediately before and after (see Fig 1A). To examine the effects of task and recording location, we conducted an analysis of variance (ANOVA) with those 2 factors, comparing task versus nontask periods at hippocampal, temporal lobe, and amygdala recording locations. We found both a main effect of task (*F*_1, 43_ = 8.6, *p* = 0.005) and a task by recording location interaction (*F*_2, 43_ = 6.2, *p* = 0.02; see Fig 1B). Subsequent analysis indicated that these results were driven by an increase in IIS frequency in the hippocampus during task versus nontask periods (*t*_8_ = 2.9, *p* = 0.005), but not in the temporal lobe or amygdala (both *p* > 0.13). In addition, we compared the task‐related increase in hippocampal IIS frequency between patients with and without a temporal lobe focus and with electrodes in left and right hemisphere, but did not find a significant difference in either case (both *p* > 0.13).

Next, we compared encoding and retrieval periods within the spatial memory task to identify whether either memory operation was primarily responsible for the increase in IIS frequency. However, we found no differences between IIS frequency at encoding or retrieval in the hippocampus (*p* > 0.29; see Fig 1C). Similarly, we found no evidence for a relationship between IIS frequency and task performance in any region (all *p* > 0.53).

### *IIS Frequency Is Increased in the Hippocampus and Temporal Lobe during an Episodic Memory Task*

Next, we compared normalized IIS frequency during the episodic memory task with nontask periods of approximately equal duration (see Fig 2A). An ANOVA with the same 2 factors again revealed a main effect of task (*F*_1, 43_ = 12.2, *p* < 0.001) and task by recording location interaction (*F*_2, 43_ = 5.6, *p* = 0.02; see Fig 2B). Subsequent analysis indicated that these results were driven by an increase in IIS frequency during task versus nontask periods in the hippocampus (*t*_10_ = 3.2, *p* = 0.002) and temporal lobe (*t*_11_ = 2.4, *p* = 0.02), but not the amygdala (*p* = 0.58). Again, task‐related increases in IIS frequency did not differ according to temporal lobe focus or implanted hemisphere (both *p* > 0.56).

Next, we asked whether this task‐related increase in IIS frequency was driven by encoding or retrieval specifically. Interestingly, an ANOVA with factors of encoding and retrieval, and hippocampal and temporal lobe recording site revealed a main effect of task phase (*F*_1, 44_ = 21, *p* = 0.001), with increased IIS frequency in both the hippocampus and lateral temporal lobe during encoding, but no effect of recording site or interaction (both *p* > 0.13; see Fig 2C). Again, we found no evidence for a relationship between IIS frequency and recognition or associative memory performance during either encoding or retrieval (all *p* > 0.12).

### *Temporal Lobe Interictal Spike Frequency Is Not Modulated by an Attentional Bias Task*

Finally, we compared normalized IIS frequency during the attentional bias task with nontask periods of approximately equal duration immediately before and after (see Fig 3A). However, an ANOVA with factors of task versus nontask and recording location revealed no main effects or interaction (all *p* > 0.54; see Fig 3B). In addition, we made a within‐subjects comparison of IIS frequency changes on hippocampal and temporal lobe contacts during the episodic memory and attentional bias task, for those patients who completed both. This revealed a main effect of task (*F*_1,23_ = 6.6, *p* = 0.04), driven by a significantly greater IIS frequency increase during the episodic memory task, but no other main effects or interactions (both *p* > 0.88; see Fig 3C).

## Discussion

We have shown that IIS frequency increases in the hippocampus during the performance of a spatial memory task, which is known to engage that region in both rodents^6^ and humans.^3^, ^7^ Similarly, we have shown that an episodic memory task increases IIS frequency in both hippocampus and lateral temporal lobe. These findings appears to be region specific, as task‐related changes in IIS frequency were not observed in the amygdala, which is not thought to be engaged by either task.^4^, ^8^ Moreover, an attentional bias task—which is not believed to be hippocampal dependent—elicited no significant change in IIS frequency within any temporal lobe region. Nonetheless, we cannot rule out the possibility that task‐dependent increases in IIS frequency resulted from other aspects of the memory tasks that were not controlled for, such as the nature of the stimuli used or the level of cognitive effort required.

Although IISs have been proposed to negatively correlate with performance in visual recognition and word recall tasks,^9^, ^10^ task‐related increases in spike activity have not previously been reported. This underlines the importance of removing IIS before analyzing task‐related changes in oscillatory power, as they could make a strong and task‐dependent contribution. It has previously been suggested that suppression of IISs can occur during task execution due to arousal or attention,^11^ which we controlled for by interleaving task and nontask periods, but we report the opposite pattern of results here.

Within the episodic memory task, IIS frequency was significantly higher during encoding than retrieval within both the hippocampus and lateral temporal lobe, suggesting that memory formation may cause greater engagement of those regions than later recall. Surprisingly, however, we found no relationship between IIS frequency and performance on either task. This may result from task structure, with the spatial memory task being self‐paced, and long (>6 seconds) encoding and retrieval epochs used in the episodic memory task, potentially nullifying any effect of IISs. We also found no evidence of laterality in task‐related IIS frequency increases, consistent with previous functional magnetic resonance imaging evidence demonstrating bilateral hippocampal activation during each task.^4^, ^8^

Finally, it is important to note that the patient group examined here exhibited a wide range of epilepsies, the majority with a mesial temporal origin. Despite this diversity, abnormal interictal activity was consistently observed in the same hippocampal and temporal neocortical regions, suggesting that the functional networks mediating spatial and episodic memory formation can be appropriated by abnormal epileptiform activity. In summary, spatial and episodic memory function promotes regional interictal spiking, suggesting that spike activation can reflect network engagement by cognitive tasks in epilepsy.

## Author Contributions

U.V., D.B., J.A.B., N.B, and M.C.W. contributed to the conception and design of the study. U.V., D.B., J.A.B., B.D., A.J., P.N., and R.R. contributed to the acquisition and analysis of data. U.V., D.B., J.A.B., A.J., N.B, and M.C.W. contributed to drafting a significant portion of the manuscript or figures.

## Potential Conflicts of Interest

Nothing to report.
